# Delivery of Difficult News Among Residents at a Tertiary Hospital In Kenya Using a Short Training Video (UNMASKES)

**DOI:** 10.15694/mep.2021.000122.1

**Published:** 2021-05-12

**Authors:** Karishma Sharma, Jasmit Shah, Zohray Talib, Sayed Ali

**Affiliations:** 1 Aga Khan University; 2 California University of Science and Medicine

**Keywords:** Delivery of difficult news, residents, effective communication, sub-Saharan Africa.

## Abstract

This article was migrated. The article was marked as recommended.

Background

Delivery of difficult news (DDN) remains a challenging task for even the most experienced of providers. Little has been studied about delivery of difficult news among resident physicians in sub-Saharan Africa (SSA). We developed a 4-minute, graphic video using the acronym UNMASKES to help improve delivery of difficult news between resident physicians and their patients.

Objective

To determine the impact of the UNMASKES training video in improving resident communication when delivering difficult news.

Methods

We conducted a prospective study amongst all residents at the Aga Khan University hospital in Nairobi, Kenya and Dar es Salaam, Tanzania from February to September, 2019. After completing a pretest survey, residents received a one-hour comprehensive training on delivery of difficult news using UNMASKES. A link to the UNMASKES video was provided to the residents for real-time reference. Post-test surveys were completed at 4 and 12 weeks respectively.

Results

A total of 102 (68%) residents completed the surveys. At 12 weeks, we found that residents improved in 6 key areas; notified their patients before delivering difficult news, ensured a private and quiet room, provided information in small amounts to patients and family members, provided a summary after delivery of difficult news, followed up with patients at 24-48 hours after delivering difficult news, and felt better prepared to deal with patient and family emotions when delivering difficult news.

Conclusions

UNMASKES, a 4-minutes, easily accessible, graphic video showed promising results in training our residents to foster effective communication while delivering difficult news to their patients.

## Introduction

Effective communication remains a cornerstone to a successful physician-patient relationship ( Stewart, 1995). Parvaiz Koul termed effective communication as at ‘the heart of art of medicine’ and also recognized that within developing countries, with inefficient health care systems, the emphasis to foster better communication is lacking ( Koul, 2017). Surprisingly, only 7% of communication is verbal and therefore communication is not only about what we say, but also how we relay this information ( Pellegrini, 2017). Patient surveys have repeatedly reflected on the need for better communication from medical providers and it is not unusual for medical providers to over-estimate their ability to communicate to patients ( Stewart, 1995). It has been shown that medical providers and patients often disagree on what transpires when delivering difficult news and this seems to be more evident in matters related to patient emotions and privacy on personal matters ( Toutin-Dias, Daglius-Dias and Scalabrini-Neto, 2018). Furthermore, there seems to be discrepancy on perceptions of effective communication between medical providers and patients ( Tongue, 2005). Arora *et al.* described the three goals to effective communication as: establishing good interpersonal relationship, encouraging effective information exchange and allowing for shared decision making ( Arora, 2003).

Despite well-known benefits of early initiation of difficult news conversations, many barriers exist in effective patient communication especially when delivering difficult news or fostering end of life discussions ( Ha and Longnecker, 2010).Some of these barriers include, lack of physician training in effective communication skills, patient anxiety, denial or the desire to protect family members and the amount of time spent having such discussions ( Bernacki and Block, 2014).

In a study conducted by Seifart *et al.,* only 46.2% of the surveyed cancer patients were completely satisfied with how difficult news was delivered to them using the commonly used SPIKES protocol ( Seifart *et al.,* 2014). SPIKES is an acronym that stands for setting, perception, invitation, knowledge, emotions and summary. Marschollek *et al.* surveyed cancer patients to determine the SPIKE implementation, and even though implemented in a satisfactory way by oncologists, it could be improved in some key areas mainly: perception, invitation and summary ( Marschollek *et al.,* 2019). Some studies have suggested that the SPIKES acronym might not fit the needs of certain populations within their respective countries, while others have suggested adding context to the acronym to make it more specific and comprehensive for their specific populations ( Seifart *et al.,* 2014; Mirza *et al.,* 2019). Kayrouz and colleagues added a cultural formulation interview to the SPIKES protocol to better communicate mental health diagnosis to patients from culturally and linguistically diverse backgrounds ( Kayrouz, Senediak and Laube, 2017).

Schmit *et al.* reported that more than eighty percent of residents received no formal classroom teaching on delivering difficult news during their residency despite frequently delivering difficult news ( Schmit *et al.,* 2016). Similarly, in a study conducted in Iran, only 13.6% had been taught to deliver bad news ( Arbabi *et al.,* 2010). Geeta *et al.* reported that only 16% of final pediatric resident in one institution, had received any formal training in delivering difficult news, while Narayan *et al.* reported that 83.6% of radiology resident at two urban residency program reported no training in delivering difficult news to their patients ( Geeta and Krishnakumar, 2017; Narayan *et al.,* 2018). In addition, such discussions were common and often unsupervised ( Schmit *et al.,* 2016).

In sub Saharan Africa, the literature on effective communication remains sparse and the barriers to delivery of difficult news remain largely unknown. A study done in Nigeria showed only 22% of health care provider had received some form of training in breaking bad news and reported better competency at breaking bad news. However, the same study also noted that very few provided a private place for disclosure of bad news and were seldom accompanied by a nurse ( Adebayo *et al.,* 2013).LaVigne *et al.* showed that 100% of physician, nurse and hospice staff surveyed from various institutions in Botswana, felt that education remained a major obstacle to further expansion of palliative care services ( LaVigne *et al.,* 2018).

Teaching of effective communication largely occurs through observation in clinical settings with junior physicians usually refining their skills over time ( Colletti *et al.,* 2001). The first and the most widely used acronym, SPIKES, was developed in the 1990’s by an oncology group ( Baile *et al.,* 2000). Several other acronyms have been developed including ABCDE and BREAKS ( Narayanan, Bista and Koshy, 2010; Konstantis and Exiara, 2015). Some studies have actually called for a modification to these acronyms to better suit their specific populations ( Seifart *et al.,* 2014). Furthermore, in an era of smartphones and dynamic technology, many of these acronyms, lack the visual clues and accessibility to help the physicians recall how to deliver important news to their patients. None of these acronyms have been studied in our setting.

## Methods

Based on the results from our study published earlier looking at delivering difficult news in our institution and the need to better train our residents ( Sharma, Shah and Ali, 2019), we developed a novel and short; less than four minutes, high resolution, graphic video using the acronym UNMASKES which stands for: Utilize, Notify, Minimize, Acknowledge, Strategize, Knowledge, Empathize and Summarize. UNMASKES, was initially developed using key communication components specific to breaking bad news to patients and from other older acronyms. The UNMASKES video ( https://youtu.be/3hA3OmbURf8) shows and discusses the various communication steps to help improve delivery of difficult news to families and patients. The video is simple and self-explanatory, therefore can be used by all healthcare workers irrespective of previous training in delivering difficult news. With the evolution, convenience and availability of smart phones to medical learners, the UNMASKES training video, unlike older acronyms, is readily available, on demand to medical learners for review and has the potential to better enhance the learning experience in our setting. SPIKES and other protocols unfortunately lack the visual clues and can be cumbersome to remember for medical providers. In addition, with the existing pandemic affecting the globe and with limited time available to train healthcare workers, this video can potentially be used in settings with limited palliative care support. The advantages of UNMASKES is that it is available on demand, visually appealing and can be quickly viewed by any resident or physician before delivering difficult news to patients. A link to the video has been made available in the appendix section of the paper.

We conducted a prospective observational study from February to September, 2019, with a pre- and post-intervention assessment among residents currently enrolled in a Graduate Medical Education (GME) program at the Aga Khan University Hospital, Nairobi (AKUHN) and Dar es Salaam. Inclusion criteria were all residents currently enrolled in all GME program at both institutions irrespective of year of study that provided informed consent. Ethical approval to conduct this study was obtained from AKUHN, Ethical Review Committee. Participation in this study was fully voluntary and the participants had the right to withdraw their participation from the study at any point with no consequences. Confidentiality was maintained at every point of data collection and analysis. Data and information collected was made known to only the investigators, to maintain confidentiality.

We developed a semi-structured pre-test and post-test survey from questions used in previous studies. The survey was reviewed by a team of palliative care team members for content and a team of medicine faculty and nursing staff for ease of language and clarity. The questions were aimed at assessing the resident’s experience and proficiency as well as identifying challenges. Both the pre-test and post-test surveys are available as a supplementary file (see Supplementary File 1). After obtaining written consent, a pre-test survey was sent out to all residents from all the departments. After completion of the pre-test survey, the UNMASKES video was introduced to the residents during a mandatory departmental one-hour training period. The training took place physically at both the institutions, and all residents were required to attend the meeting. Residents had an opportunity to view the video, discuss implementation of all stages and help address any questions. A reminder and a link to the video was then disseminated to the residents on a weekly basis. A post-test survey was then conducted at 4 and 12 weeks. The pre and post survey data was collected through Research Electronic Data Capture - REDCap (web interface of a research electronic data capture instance, powered by Vanderbilt and supported in part by the National Institute of Health ( Harris *et al.,* 2009). The data was de-identified for analysis. Categorical data has been presented as frequencies and percentages whereas continuous data is presented as means and standard deviations. Continuous data has been tested for normality using the Shapiro Wilks tests. Univariate analysis has been conducted using Chi-square test or Fisher’s exact test for categorical data and Student’s t-test or Mann Whitney test for continuous data separately for the pre-test and post-test surveys.

## Results/Analysis

All 150 residents from nine residency programs in Nairobi and three residency programs in Dar es Salaam were asked to complete the online pre-test survey. One hundred and two (102) residents consented to participate in the twelve-week study (response rate 68%). An approximate equal representation of residents across gender and year of study was seen in the study. Of all the participants, ninety-four percent (94%) reported delivering difficult news during their medical practice. The Baseline characteristics and the results from the initial survey were published as a letter to the editor in the Journal of pain and symptom management ( Sharma, Shah and Ali, 2019).

At four weeks, the post-test survey showed that seventy-five percent (75%) of residents had delivered difficult news to their patients and a majority of them (60%) delivered the news a total of one to five times a week. Eighty-seven percent (87%) of residents found the UNMASKES video very helpful in helping them deliver difficult news to their patients. Statistical differences between baseline and four weeks found are summarized in Table 1. From the 10 components of the UNMASKES approach, residents demonstrated improvement in 6 at both at 4 weeks and 12 weeks. Residents were more likely to notify patients prior to delivering difficult news (47.8% at baseline vs 62.2% at four weeks, p = 0.019) and to provide information in small amounts to their patients (62.2% at baseline vs 85.7% at four weeks, p<0.001). Residents consistently verified patient knowledge of disease process prior to delivering difficult news (87.8% at baseline vs 100% at four weeks, p = 0.022) and they ensured a private and quiet room to hold such conversations (81.1% at baseline vs 93.5% at four weeks, p = 0.022). They were able to better handle the patient and family emotions during and after delivering difficult news (32.2% at baseline vs 62.3% at four weeks, p<0.001) and ensured follow up with patients at 24-48hours after delivering difficult news (16.7% at baseline vs 39% at four weeks, p = 0.002).

At twelve weeks, post-test survey results showed that seventy-eight percent (78%) of the residents had delivered difficult news with sixty-one percent (61%) delivering news one to five times a week. Statistical differences between baseline and twelve weeks are summarized in Table 2. Residents at twelve weeks were more likely to notify patients prior to delivering difficult news (47.8% at baseline vs 84.6% at twelve weeks, p<0.001), provide information in small amounts (62.2% at baseline vs 92.3% at twelve weeks, p<0.001), provide a summary to their patients (66.7% at baseline vs 96.2% at twelve weeks, p<0.001), follow up with their patients after 24-48 hours (16.7% at baseline vs 52.6% at twelve weeks, p<0.001) and were better able to handle patient and family emotions (32.2% at baseline vs 69.2% at twelve weeks, p<0.001). Residents also ensured a private and quiet room to hold such conversations (81.1% at baseline vs 94.7% at twelve weeks, p<0.001). Use of simple terminology, discussion with other health care professional and checking how much the patient knows during the process of delivering news remained unchanged at the end of the twelve weeks.

Nine out of ten residents reported that they would use the UNMASKES video in the future. The residents highlighted the following key benefits to the UNMASKES video: short, concise, structured, graphic and available on demand.

**Table 1:** Comparison between baseline and four weeks with the respective p values.

Variable		Baseline		4 weeks		p value
		N	(%)	N	(%)	
Times Deliver Difficult News	None	12	11.8%	25	24.5%	0.089
	1-5	70	68.8%	63	61.8%	
	6-10	12	11.8%	10	9.8%	
	>10	8	7.8%	4	3.9%	
Notify Patient	Yes	43	47.8%	51	66.2%	0.019
	No	47	52.2%	26	33.8%	
Discuss with other medical professionals	Yes	52	57.8%	50	64.9%	0.426
	No	38	42.2%	27	35.1%	
Ensure a Private and Quiet Room	Yes	73	81.1%	72	93.5%	0.022
	No	17	18.9%	5	6.5%	
Introduce Yourself to Family	Yes	90	100.0%	76	100.0%	0.461
	No	0	0.0%	1	0.0%	
Check How Much Patient Knows	Yes	79	87.8%	75	100.0%	0.022
	No	11	12.2%	2	0.0%	
Use Simple Terminology	Yes	89	98.9%	76	100.0%	1
	No	1	1.1%	1	0.0%	
Patients and Family Emotions	Yes	29	32.2%	48	62.3%	<0.001
	No	61	67.8%	29	37.7%	
Small Amounts Information	Yes	56	62.2%	66	85.7%	<0.001
	No	34	37.8%	11	14.3%	
Summary Given	Yes	60	66.7%	61	79.2%	0.083
	No	30	33.3%	16	20.8%	
Follow up after 24 - 48 hrs.	Yes	15	16.7%	30	39.0%	0.002
	No	75	83.3%	47	61.0%	

**Table 2:** Comparison between baseline and 12 weeks with the respective p values.

Variable		Baseline		12 weeks		p value
		N	(%)	N	(%)	
Times Deliver Difficult News	None	12	11.8%	24	23.5%	0.017
	1-5	70	68.8%	48	47.1%	
	6-10	12	11.8%	18	17.6%	
	>10	8	7.8%	12	11.8%	
Notify Patient	Yes	43	47.8%	66	84.6%	<0.001
	No	47	52.2%	12	15.4%	
Discuss with other medical professionals	Yes	52	57.8%	53	63.3%	0.203
	No	38	42.2%	25	36.7%	
Ensure a Private and Quiet Room	Yes	73	81.1%	76	97.4%	0.001
	No	17	18.9%	2	2.6%	
Introduce Yourself to Family	Yes	90	100.0%	78	100.0%	NA
	No	0	0.0%	0	0.0%	
Check How Much Patient Knows	Yes	79	87.8%	73	93.3%	0.174
	No	11	12.2%	4	5.3%	
Use Simple Terminology	Yes	89	98.9%	78	100.0%	1
	No	1	1.1%	0	0.0%	
Patients and Family Emotions	Yes	29	32.2%	54	69.2%	<0.001
	No	61	67.8%	24	30.8%	
Small Amounts Information	Yes	56	62.2%	72	92.3%	<0.001
	No	34	37.8%	6	7.7%	
Summary Given	Yes	60	66.7%	75	96.2%	<0.001
	No	30	33.3%	3	3.8%	
Follow up after 24 - 48 hrs.	Yes	15	16.7%	41	52.6%	<0.001
	No	75	83.3%	37	47.4%	

## Discussion

Similar to other studies more than fifty percent reported having received no formal training in delivering difficult news during their formative years of medical education ( Arbabi *et al.,* 2010; Adebayo *et al.,* 2013; Geeta and Krishnakumar, 2017).

Four and twelve weeks post implementation of UNMASKES, we found that our residents were more proficient in key elements of effective patient communication related to delivering difficult news. Specifically, they were more likely to notify patients prior to delivering difficult news, they were better in providing information in small amount to their patients and consistently verified patient knowledge of disease process prior to delivering difficult news. Our residents also ensured a private and quiet room when delivering difficult news to their patients and were able to better handle the patient’s and family emotions during and after delivering difficult news. Our residents also ensured follow up with patients at 24-48hours after delivering difficult news. Interestingly, we found that at 12 weeks, our residents either maintained or improved each measurable variable in delivery of difficult news highlighting the potential success of UNMASKES in training residents in our setting to improve their overall communication skills.

In our previous study, we found that over 80% of our residents reported significant anxiety prior to delivering difficult news to their patients ( Sharma, Shah and Ali, 2019). Dosanj *et al.* in their study also reported that residents expressed fear and stress dealing with family member reaction to delivery of bad news ( Dosanjh, Barnes and Bhandari, 2001). Jameel and colleagues similarly reported that 83% of residents surveyed in three teaching hospital in Peshawar, Pakistan were either not or somewhat comfortable delivering difficult news to the patients before their intervention ( Jameel, Noor and Ayub, 2012). In a study by Lee and Yi, 90% of resident and fellows in a teaching hospital reported more than average level of stress when breaking bad news to their patients, while 30% reported a bad experience due to improperly delivered bad news to a patient ( Lee and Yi, 2013). A recent study done in South Africa among health care workers found that they feel emotionally burdened by patient and family expectations because of lack of communication skills to deal with the vast needs of the patients ( Ganca *et al.,* 2016). We found that at four and twelve week respectively, using UNMASKES, our residents felt better prepared to deal with patient and family emotions after delivering difficult news.

UNMASKES to-date remains the first developed, short, concise and self-explanatory video that medical providers can access at any time and especially prior to delivering difficult news. The video requires minimal training, is simple to follow and remains a cost effective method at delivery of difficult news especially in SSA with scarce resources. Using UNMASKES, we saw a significant improvement in delivery of difficult news among residents at our institution at 4 and 12 weeks post implementation.

Despite the favorable results from this study, there are a number of limitations to using UNMASKES. UNMASKES is available only in English and attempts are underway to translate it into the local language. UNMASKES is also available via the internet, which may be unavailable or unaffordable in some parts of the country. Our study was limited to our residents only and did not include other healthcare workers or residents from other local institutions. In addition, our study was limited to 12 weeks and hence long-term effects of the intervention is currently unknown.

## Conclusion

UNMASKES, a four minutes, easily accessible, colorful and graphic video showed promising results in training our residents to foster effective communication while delivering difficult news to their patients. Further studies need to be performed to assess the long-term applicability of this protocol.

## Take Home Messages

• UNMASKES, a novel 4-minute graphic video, showed promising results in fostering better delivery of difficult news among residents at our institution.
• This is the first every such study conducted in sub-Saharan Africa around delivery of difficult news to patients and their families.
• Further studies are required to determine the long term effects of UNMASKES in delivering of difficult news to patients.

## Notes On Contributors

Karishma Sharma, MBChB, is a senior resident within the Department of Medicine and is completing her Master of Medicine at Aga Khan University, Nairobi. She has a keen interest in improving end of life communication amongst medical trainees.

Jasmit Shah, Ph.D. is a biostatistician within Department of Medicine and the Chair of the Department Research Committee at Aga Khan University, Nairobi. He help teach research methodology to medical residents at the University. ORCiD: https://orcid.org/0000-0002-7957-5426

Zohray Talib, MD is a senior Associate Dean with the Academic Affair office at California University of Science and Medicine. She is also the Chair of Medical Education at the same school. One of her interest lies in fostering better education opportunities, via collaborations, in sub Saharan Africa.

Sayed K. Ali, MD, FACP is an Associate Professor in the Department of Medicine. He is also a faculty in internal medicine and palliative care within the department. His interest lies in fostering better physician-patient communication. ORCiD: https://orcid.org/0000-0003-0750-2903

## Appendices

### APPENDIX I:

UNMASKES VIDEO

The video is currently available on the following link: https://youtu.be/3hA3OmbURf8
