# Supplementary material for: Delivery of Difficult News Among Residents at a Tertiary Hospital In Kenya Using a Short Training Video (UNMASKES)
Source: MedEdPublish (2016). 2021 May 12;10:122. [Version 1] doi: 10.15694/mep.2021.000122.1 (PMC10939531; doi:10.15694/mep.2021.000122.1)
Supplement: Peer review history

# Reviewer report: Reviewer response for version 1

**DOI:** 10.21956/mep.18950.r27117

**Published:** 2021-10-11

**Authors:** Gert Van Zyl

**Affiliations:** 1 University of the Free State

This review has been migrated. The reviewer awarded 4 stars out of 5 Thank you for sharing this informative research on a very important topic. The video as tool called UNMASKED is very helpful and enabling to help to guide students to master the process in delivering difficult news. I did wonder what the outcome would be in a more global context and if the same high impact will be measured. Still worth watching although it focusses more on end-of-life news compared to more generic approach to bad news.

Reviewer Expertise:

NA

No decision status is available

# Reviewer report: Reviewer response for version 1

**DOI:** 10.21956/mep.18950.r27118

**Published:** 2021-07-16

**Authors:** Richard Hays

**Affiliations:** 1 James Cook University

This review has been migrated. The reviewer awarded 3 stars out of 5 If what this article claims is true - that attending a 1 hour workshop and having available a 4 minute video clip a for refreshing has a significant effect on behaviour 4 and 12 weeks later - this would be amazing. The topic is of course important and is included in the curriculum of most medical schools I have visited. The video focuses on end of life care, which is perhaps only a small part of delivering bad news. I found it rather complicated but if it is useful to learners, it is an appropriate framework to use. Addressing the following questions may merit an extra star:- using 'new' acronyms like DDN and SSA should be avoided to improve readability- it is not clear which results come from self-report surveys or observation by others.- more details of the observation are needed. How was it done, was there any pre-workshop observation, how were observers trained, how were data analysed?

Reviewer Expertise:

NA

No decision status is available
